# Sleep Quality, but Not Personality Traits, Mediates the Relationship between Chronotype and Life Satisfaction: A Study in Young Adults

**DOI:** 10.3390/clockssleep6030022

**Published:** 2024-07-23

**Authors:** Anat Lan, Yelena Stukalin, Haim Einat

**Affiliations:** School of Behavioral Sciences, Tel-Aviv-Yaffo Academic College, Tel-Aviv 6818211, Israelhaimh@mta.ac.il (H.E.)

**Keywords:** chronotype, circadian disruption, sleep quality, life satisfaction, personality traits, well-being

## Abstract

Chronotype reflects the morningness–eveningness preference over a 24 h period. Significant data indicate meaningful differences between evening types (ETs) and morning types (MTs) in behavior, personality traits, health, and well-being. This study explores the interactions between chronotype, sleep, personality, and life satisfaction among 254 undergraduate college students (mean age 23.79 ± 1.85). Using online questionnaires, the participants provided demographic information and completed assessments, including the Morningness–Eveningness Questionnaire (MEQ), the Pittsburg Sleep Quality Index (PSQI), a shortened version of the Big Five Inventory (BFI-10), and a life satisfaction uniscale measure. The results revealed a significant association between chronotype and both life satisfaction and sleep quality, where ETs exhibited poorer outcomes compared to MTs. Additionally, the chronotype correlated with agreeableness and conscientiousness, with later chronotypes linked to reduced scores in these personality traits. A key finding in this study was revealed in a mediation analysis in which sleep quality was found to mediate the relationship between chronotype and life satisfaction. The mediation analysis highlighted sleep quality as a crucial process connecting chronotype to life satisfaction. The findings emphasize the importance of addressing sleep quality in interventions aimed at enhancing life satisfaction and overall well-being among ETs. Overall, our results provide valuable insights into the intricate relationships between chronotype, personality, sleep quality, and subjective well-being.

## 1. Introduction

Circadian rhythms play a pivotal role in a broad array of physiological and behavioral processes and functions [1]. The human master clock located within the suprachiasmatic nucleus (SCN) of the hypothalamus regulates our daily rhythms, synchronizing environmental cues such as light, temperature cycles, and nutritional intake with its self-sustained internal oscillator [2]. An important factor in relation to circadian rhythms is the individual preference for sleep–wake patterns over a 24-hour period, referred to as the morningness–eveningness preference. Individuals identified as morning types (MTs), also known as “larks”, exhibit a preference for early morning awakenings and the corresponding early evening bedtimes, whereas evening types (ETs), or “owls”, demonstrate a propensity for later morning wake-ups (even extending to the afternoon) and a preference for going to sleep late at night or in the early morning. Individuals displaying sleep–wake patterns between these two extremes are categorized as intermediate types on this behavioral continuum [3]. The chronotype represents a trait indicating the inclination towards morningness or eveningness preferences [4]. In the general population, the distribution of morningness–eveningness preferences follows a nearly normal distribution, with a slight bias towards eveningness [3,5].

The differences between MTs and ETs extend beyond sleep and wake timings, encompassing a range of behavioral and psychological traits as well [6]. A later chronotype was associated with sleep deprivation, lower sleep quality, reduced perceived quality of life and life satisfaction, and an increased risk for mood disorders and depression [7,8,9,10,11,12,13]. A plausible explanation for these effects is the necessity for evening-preference individuals to conform to the conventional morning-oriented social schedule, whether it pertains to school or work hours. Such adaptation disrupts their inherent preferences and frequently results in chronic sleep deficiency [7]. Both desynchronization of the internal clock and sleep deprivation were demonstrated to have significant negative consequences on life [7,14,15].

The association between chronotype and personality traits have also been documented in the literature. Using the Big Five model, which comprises five traits, extraversion, agreeableness, conscientiousness, neuroticism, and openness, researchers have explored these associations in depth. One trait that was consistently demonstrated to have a positive relationship with morningness across studies is conscientiousness [6,16]. As for other personality traits, the data are incongruent, where some studies show relationships and some do not. For instance, a few studies found higher neuroticism in ET individuals [17,18,19,20] while others found no relationship [21,22,23,24]. Similarly, some show that ET individuals have higher openness [19,23], but others did not identify such a relationship [17,18,20,21,22,24]. 

Though there are data regarding the relationship between chronotypes and sleep, chronotypes and personality, and chronotypes and well-being and life satisfaction, we did not find any work that attempts to explore all these factors in one matrix. Hence, the primary objective of this study was to explore the relationship between chronotype and perceived life satisfaction among students, with attention to the additional factors of sleep and personality traits. We aimed to investigate whether an association between chronotypes and life satisfaction can be mediated by either sleep quality or the Big Five personality factors or both. Our central hypothesis posited a positive correlation between morningness and life satisfaction, with sleep quality and personality traits playing a mediating role in this relationship. 

## 2. Results

### 2.1. Descriptive Statistics and Demographics

The demographic data are presented in Table 1. The means and standard deviations for all the variables are presented in Table 2. The distribution of chronotypes, as assessed by the MEQ questionnaire, was as follows: morning types (*n* = 56), intermediate type (*n* = 126), and evening types (*n* = 72). The mean score for sleep quality, measured using the PSQI questionnaire, was 5.57. Notably, a score above 5 is typically considered to indicate sleep disturbance [25]. In our sample, 119 out of the 254 students received scores above 5, suggesting impaired subjective sleep quality. Regarding life satisfaction, measured by the QOL uniscale, the mean was 75.40. This is substantially higher than the scale midpoint of 50, indicating that overall the participants rated their satisfaction with life quite favorably. 

### 2.2. Chronotype, Sleep Quality, Life Satisfaction, and the Big Five Personality Factors

The chronotype (MEQ score) demonstrated a positive significant correlation with sleep quality (r = −0.33, *p* < 0.001), life satisfaction (r = 0.3, *p* < 0.001), conscientiousness (r = 0.31, *p* < 0.001), and agreeableness (r = 0.18, *p* = 0.004). Morning types (MTs), compared to evening types (ETs), reported higher levels across all these variables. Aside from chronotype, life satisfaction was negatively correlated with sleep quality (r = −0.35, *p* < 0.001) and neuroticism (r = −0.21, *p* < 0.001) and positively correlated with agreeableness (r = 0.16, *p* = 0.012) and conscientiousness (r = 0.15, *p* = 0.015). The correlations table is presented in Table 3.

### 2.3. Mediation Analyses

After establishing a significant link between chronotype and life satisfaction, we aimed to investigate potential factors that could mediate this relationship. We considered three candidate mediators: 1. sleep quality, 2. agreeableness, and 3. conscientiousness. Since there were no significant associations between chronotype and neuroticism, openness, and extraversion, these variables were not examined as potential mediators in the study. 

**Figure 1:**
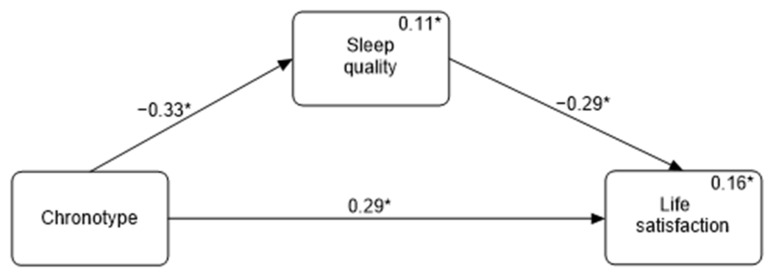
Mediation analysis depicting the relationship between chronotype and life satisfaction mediated by sleep quality. * *p* < 0.05.

In a mediational model involving sleep quality as a mediator, it was found that sleep quality mediated the association between chronotype and life satisfaction. The path coefficient from chronotype to sleep quality was statistically significant (β = −0.33, *p* < 0.01), indicating that morningness was associated with better sleep quality. The path coefficient from sleep quality to life satisfaction was also statistically significant (β = −0.29, *p* < 0.01), suggesting that sleep quality has a direct impact on life satisfaction. The indirect effect of chronotype on life satisfaction through sleep quality was significant (β = 0.11, 95% confidence intervals: 0.05–0.19), confirming that sleep quality partially mediates this relationship (Figure 1).

Remarkably, upon excluding the participants who scored above 5 on the PSQI questionnaire and focusing solely on ‘good sleepers’ (with the following sample sizes for ‘good sleepers’ in our study: 41 early chronotypes (ETs), 70 intermediate chronotypes (ITs), and 24 late chronotypes (LTs)), the association between chronotype and life satisfaction no longer remained statistically significant [F(2132) = 1.25, *p* = 0.29].

In the mediation analyses investigating whether agreeableness and conscientiousness serve as mediators, the results indicated that these factors did not mediate the relationship between chronotype and life satisfaction. For both variables, the estimated indirect effect was not significant.

Additionally, to examine the differences between chronotype groups, the MEQ score was transformed into a categorical variable using established cutoffs: evening types (ETs) (16–41), intermediate types (ITs) (42–58), and morning types (MTs) (59–86). A one-way ANOVA revealed that chronotype was significantly associated with agreeableness [F(2251) = 4.79, *p* = 0.009] and conscientiousness [F(2251) = 7.97, *p* < 0.001], but not extraversion, neuroticism, or openness. Post hoc analyses uncovered lower agreeableness scores in ETs compared to both MTs (*p* < 0.001) and ITs (*p* < 0.001). For conscientiousness, significant differences were found between all chronotype groups, with MTs showing the highest and ETs showing the lowest mean scores (*p* < 0.001).

A significant effect of chronotype was also found for sleep quality [F(2,51) = 13.941, *p* < 0.0001], with ETs showing poorer sleep quality compared to both MTs (*p* < 0.0001) and ITs (*p* < 0.001). Furthermore, chronotype was associated with life satisfaction [F(2251) = 9.034, *p* < 0.0001], with ETs exhibiting lower satisfaction relative to MTs (*p* < 0.0001) and ITs (*p* = 0.001). Table 4 summarizes the differences across chronotype groups in personality traits, sleep quality, and life satisfaction. Overall, the results demonstrate significant differences between chronotypes in sleep quality, life satisfaction, agreeableness, and conscientiousness.

### 2.4. Potential Covariates

As for potential co-variates, gender was found to be associated with chronotype, χ2 (2, N = 254) = 8.04, *p* = 0.018, with a higher percentage of men (45.5%) classified as ETs compared to women (24.8%) (Table 5). However, there was no association between gender and life satisfaction [F(1252) = 0.007, *p* = 0.93]. Similarly, living conditions and work status were not associated with life satisfaction [F(3250) = 0.19, *p* = 0.90 for living condition; [F(2251) = 2.1, *p* = 0.12 for work status].

## 3. Discussion

The primary objective of this study was to examine the relationship between chronotype and life satisfaction in students and to test whether sleep quality or any of the Big Five personality factors mediate this association. It was hypothesized that compared to evening types (ETs), morning types (MTs) will report better sleep quality and higher life satisfaction. Furthermore, we anticipated that the link between chronotype and life satisfaction would be mediated by sleep quality. Regarding the Big Five personality factors, given the inconsistent findings in the literature and the absence of clear patterns [6,16], we aimed to explore which dimensions of the Big Five are associated with chronotype and whether these associated factors act as mediators in the relationship between chronotype and life satisfaction.

The results indicated a significant association between chronotype and both life satisfaction and sleep quality. Individuals with a later chronotype (ETs) reported poorer sleep quality and life satisfaction compared to those with earlier chronotypes (MTs). Furthermore, chronotype was related to the personality traits of agreeableness and conscientiousness, such that a later chronotype was linked to reduced scores in agreeableness and conscientiousness. 

A key finding in this study was that sleep quality mediated the relationship between chronotype and life satisfaction. This suggests that poor sleep quality is a mechanism linking chronotype to overall well-being. Notably, when excluding the participants categorized as ‘bad sleepers’ (defined as those with PSQI scores above 5) and analyzing only ‘good sleepers’ (scores of 5 or below), the association between chronotype and life satisfaction was no longer significant. This implies that the role of sleep quality is crucial in shaping the link between chronotype and life satisfaction. Overall, the mediation analysis highlights sleep quality as a critical intermediary factor connecting chronotype to subjective well-being.

One potential explanation for the link between chronotype and sleep quality is that ETs often experience a misalignment between their internal biological rhythms and societal schedules. This mismatch requires ET individuals to adjust to the prevailing morning-oriented social timetable related to school or work hours. Such adaptation leads to consistent disruption of their circadian rhythm, resulting in chronic sleep deprivation and decreased sleep quality [7,26]. Furthermore, compared to MTs and ITs, ET individuals may be less exposed to daylight during waking hours, and this insufficient light exposure might lead to poorer sleep quality. Indeed, studies have indicated that ET individuals have lower light exposure during daytime [27] and that greater light exposure during the day positively influences sleep quality at night [28]. Further support for this possibility comes from research in diurnal model animals, where it was repeatedly demonstrated that reduced exposure to natural light resulted in significant disruptions to sleep/wake rhythms, which were accompanied by the emergence of multiple pathologies [29].

The factors resulting in poorer sleep quality among ETs may in turn lead to lower perceived life satisfaction. The relationship between sleep and well-being, including life satisfaction specifically, is well established [30,31,32]. A recent meta-analysis demonstrated that improving sleep leads to better mental health [33], suggesting that sleep causally affects well-being. Therefore, given the mediation findings showing sleep quality’s crucial role in the chronotype–life satisfaction link, future research should explore interventions to improve ET individuals’ sleep as a promising means to enhance their life satisfaction and overall well-being.

In our research, although the Big Five factors did not mediate the link between chronotype and life satisfaction, we did identify correlations between life satisfaction and three personality factors: agreeableness, conscientiousness, and neuroticism. This finding aligns with previous studies, which also reported a negative correlation between life satisfaction and neuroticism, as well as positive correlations between life satisfaction and agreeableness and conscientiousness [34]. However, these prior studies additionally associated life satisfaction with extraversion, which differs from our results. A possible explanation for this discrepancy, as well as the inconsistent results in general regarding the Big Five factors, could be the use of different versions of the Big Five Inventory (BFI). While the aforementioned authors utilized either a 60- or a 44-item version of the Big Five, our study employed a shorter 10-item version of the BFI (BFI-10). To the best of our knowledge, the 10-item questionnaire was not previously used in the context of the association between personality traits and life satisfaction. It is essential to acknowledge that, despite demonstrating good validity and reliability [35], the shorter version may present a limitation in our study, as it utilizes only two items to measure each personality trait.

One major limitation of the current study is that it is heavily skewed towards women. Though we did not find differences between men and women in life satisfaction, sleep quality, or personality trait measures, the unequal sample certainly casts some doubt on the generalizability of the results. This study has certain additional limitations that should be considered when interpreting the results. First, the reliance on self-report measures introduces subjectivity and recall biases. More objective measurements, such as actigraphy or wearable devices, could complement these self-report findings, at least for some of the measures. Second, the cross-sectional nature of this study precludes the determination of the directionality or causality of the observed relationships between the variables. Longitudinal studies tracking individuals over time would better elucidate the temporal ordering and potential causal pathways linking chronotype to sleep, personality, and well-being outcomes. While the current results are informative for understanding associations, experimental manipulations are ultimately needed to ascertain the causal mechanisms. Overall, incorporating multi-modal assessments and longitudinal designs in future research can help address these limitations and provide deeper insight into the complex interplay between chronotype, sleep, personality, and life satisfaction.

While this study has certain limitations, the findings point to promising avenues for improving well-being outcomes in evening type individuals. Specifically, the results highlight the potential of sleep-focused interventions, given the crucial mediating role of sleep quality in the link between eveningness and reduced life satisfaction. Optimizing sleep timing, duration, and hygiene through customized sleep education, light therapy, cognitive behavioral therapy, and other behavioral approaches could help align the evening types’ sleep patterns with their natural circadian preferences. Additionally, increasing exposure to morning bright light may boost circadian entrainment. Such sleep-targeted strategies may not only improve sleep metrics, but also enhance subjective well-being and life satisfaction in this population. Alternatively, higher awareness of the challenges faced by individuals with evening chronotypes may influence higher education institutes and employers to develop more flexible schedules. Looking ahead with targeted interventions to support the evening types’ sleep and circadian health represents an encouraging opportunity to translate these insights into real-world applications that improve quality of life.

## 4. Materials and Methods

### 4.1. Participants

In total, 254 (n = 254) undergraduate college students (210 women, 44 men, mean ± SD for age = 23.79 ± 1.85, age range 19–34) from Tel-Aviv-Yaffo academic college (Tel-Aviv, Israel) volunteered for the current study. There were no specific inclusion or exclusion criteria, and the entire cohort of volunteers was evaluated. All the procedures were approved by the institutional ethics committee (protocol # 2021178/05), and all the participants signed an informed consent form.

### 4.2. Instruments and Tools

#### 4.2.1. General and Demographic Questions

Demographic data were collected regarding the participants’ gender, work status, and living conditions. Work status options included: not working, works under 15 h a week, works more than 15 h a week. Living conditions included: alone, with parents, with a partner, with roommates. 

#### 4.2.2. The Morningness–Eveningness Questionnaire (MEQ)

The MEQ is a self-report questionnaire used to assess individuals’ chronotypes. The MEQ comprises 19 items, both Likert-type and timescale questions, pertaining to subjective phase preferences in circadian rhythms. Scores range from 16 to 86, with higher scores indicating stronger morning preferences and lower scores indicating stronger evening preferences. The global score can be converted to a three-level scale: evening types (16–41), intermediate types (42–58), and morning types (59–86) [36]. The internal consistency of the MEQ in the student population was found to be high (α = 0.78) [37].

#### 4.2.3. Life Satisfaction and Quality of Life (QoL) Uniscale 

The uniscale is a concise assessment tool designed to gauge overall life satisfaction or quality of life (QoL) using a numerical rating scale spanning from 0 to 100. Respondents are prompted to assess their present life satisfaction, with “0” signifying the least desirable state and “100” denoting the most favorable circumstance. This measure was found to have well-established validity [38].

#### 4.2.4. The Pittsburgh Sleep Quality Index (PSQI)

The Pittsburgh Sleep Quality Index (PSQI) is a validated self-report questionnaire consisting of 19 items. It is employed to assess sleep quality and disturbances experienced by individuals in the preceding month. These items are used to compute seven component scores, which encompass sleep quality, sleep latency, sleep duration, sleep efficiency, sleep disturbances, use of sleep medications, and daytime dysfunction. The combination of these component scores yields an overarching global score for sleep quality, which ranges from 0 to 21. Higher scores on this scale are indicative of lower sleep quality, with scores exceeding 5 suggesting a suboptimal quality of sleep [25].

#### 4.2.5. The Big Five Inventory (BFI-10)

We used a short version of the Big Five Inventory (BFI-10) to assess the personality dimensions of openness to experience, conscientiousness, extraversion, agreeableness, and neuroticism. Each dimension is evaluated through a pair of items, employing a five-point Likert scale spanning from ‘strongly disagree’ (1) to ‘strongly agree’ (5). Elevated scores on the scale indicate a greater manifestation of the respective personality trait. The BFI-10 has demonstrated robust retest reliability, as well as strong evidence of both convergent and discriminant validity [35]. This version of the Big Five questionnaire was designed to offer a measurement tool that maintains substantial levels of reliability and validity while significantly reducing the time required for responses.

### 4.3. Procedure

Two hundred fifty-four students signed an informed consent form and completed online questionnaires. The participants filled out general and demographic questions, the morningness–eveningness questionnaire (MEQ), the Pittsburg Sleep Quality Index (PSQI), a short version of the Big Five personality inventory (BFI-10) and a life satisfaction uniscale measure. 

### 4.4. Statistical Analysis

Pearson’s r was used to evaluate the bivariate correlations. The mediation analyses were conducted using the PROCESS macro for SPSS Statistics 23, following the procedures recommended by Hayes. The indirect effects were evaluated using a bootstrapping resampling procedure: 5000 bootstrapped samples were drawn from the data, and bias-corrected 95% confidence intervals (CI) were used to estimate the indirect effects of each of the resampled datasets. If the 95% CI for the estimates of indirect effect does not include zero, it suggests the significant mediation at the 0.05 level [39,40]. In addition, ANOVA analysis was used to assess the differences in sleep quality, life satisfaction, and the Big Five factors between the chronotype groups: morning types, intermediate types, and evening types. Significant ANOVA results were followed by post hoc analysis.

## 5. Conclusions

This study explored the relationships between chronotype, sleep quality, and life satisfaction among students, with an additional focus on the potential mediating roles of sleep quality and the Big Five personality traits. The findings reveal that sleep quality significantly mediates the relationship between chronotype and life satisfaction, indicating that individuals with later chronotypes experience poorer sleep quality, which in turn negatively affects their life satisfaction.

Furthermore, the study identified significant associations between chronotype and personality traits, specifically agreeableness and conscientiousness, though these traits did not mediate the relationship between chronotype and life satisfaction. Despite the skewed sample and the reliance on self-report measures, the results underscore the importance of sleep quality in influencing the well-being of evening types.

Given these findings, interventions aimed at improving sleep quality, particularly for evening types, could be beneficial in enhancing their overall well-being and life satisfaction. Future research should focus on longitudinal studies and objective measurements to further elucidate the causal pathways involved. Additionally, developing flexible schedules in educational and occupational settings may help accommodate the natural circadian preferences of evening types, thereby improving their sleep quality and life satisfaction.

## Figures and Tables

**Table 1:** Demographic data.

Variable	Measure
Gender	83% women
Age (years, mean ± SD)	23.79 ± 1.85
Smoking	34% smokers
Employment status (part-time on top of school)	87% employed
BMI (mean ± SD)	22.4 ± 4.6

**Table 2:** Mean and standard deviations of all variables.

Variable Name	Mean ± SD
Chronotype (MEQ score)	48.86 ± 11.05
Sleep quality (lower scores indicate better sleep quality)	5.57 ± 2.46
Self-reported life satisfaction	75.40 ± 12.82
Big 5—Extraversion	6.94 ± 1.62
Big 5—Neuroticism	5.50 ± 1.85
Big 5—Openness to experience	7.22 ± 1.75
Big 5—Agreeableness	7.06 ± 1.60
Big 5—Conscientiousness	7.8 ± 1.59

**Table 3:** Relationship between chronotype, sleep quality, life satisfaction, and the Big Five personality factors based on bivariate correlations. * *p* < 0.05.

	Chronotype (MEQ Score)	Life Satisfaction (QOL)	Extraversion	Agreeableness	Conscientiousness	Neuroticism	Openness	Sleep Quality (PSQI)
Chronotype (MEQ Score)	---	r = 0.297, *p* < 0.001 *	r = 0.039, *p* = 0.53	r = 0.18, *p* = 0.004 *	r = 0.31, *p* < 0.001 *	r = −0.04, *p* = 0.44	r = −0.06, *p* = 0.33	r = −0.33, *p* < 0.001 *
Life Satisfaction (QOL)		---	r = 0.06, *p* = 0.28	r = 0.16, *p* = 0.012 *	r = 0.15, *p* = 0.015 *	r = −0.21, *p* < 0.001 *	r = −0.04, *p* = 0.82	r = −0.35, *p* < 0.001 *
Extraversion			---	r = 0.196, *p* = 0.002 *	r = 0.132, *p* = 0.035 *	r = 0.009, *p* = 0.89	r = −0.04, *p* = 0.50	r = −0.07, *p* = 0.248
Agreeableness				---	r = 0.09, *p* = 0.15	r = −0.268, *p* < 0.001	r = −0.01, *p* = 0.88	r = 0.06, *p* = 0.376
Conscientiousness					---	r = −0.08, *p* = 0.17	r = −0.11, *p* = 0.86	r = −0.244, *p* < 0.001 *
Neuroticism						--	r = 0.005, *p* = 0.93	r = 0.203, *p* = 0.001 *
Openness							---	r = 0.059, *p* = 0.35
Sleep Quality (PSQI)								---

**Table 4:** Differences in sleep quality, life satisfaction, and Big Five personality traits based on chronotype. MTs = morning types, ITs = intermediate types, ETs = evening types.

	Morning Types (MTs)	Intermediate Types (ITs)	Evening Types (ETs)	F	*p*-Value	Post hoc Comparisons
Sleep quality (lower scores indicate better sleep quality)	4.55 ± 2.65	5.38 ± 2.15	6.69 ± 2.41	13.94	<0.001	MT < IT, MT < ET, IT < ET
Life satisfaction	79.16 ± 10.92	76.60 ± 11.48	70.37 ± 14.89	9.03	<0.001	MT = IT, MT > ET, IT > ET
Extraversion	7.08 ± 1.55	6.92 ± 1.53	6.87 ± 1.83	0.29	0.75	
Neuroticism	5.25 ± 1.82	5.64 ± 1.86	5.46 ± 1.87	0.9	0.41	
Openness to experience	6.93 ± 1.78	7.36 ± 1.69	7.20 ± 1.80	1.21	0.3	
Agreeableness	7.52 ± 1.32	7.10 ± 1.56	6.65 ± 1.77	4.79	0.009	MT = IT, MT > ET, IT > ET
Conscientiousness	8.39 ± 1.43	7.82 ± 1.59	7.29 ± 1.56	7.97	<0.001	MT > IT, MT > ET, IT > ET

**Table 5:** Chronotype classification based on gender. The numerical values within the table represent the frequency of individuals within each respective category.

	Morning Types (MTs)	Intermediate Types (ITs)	Evening Types (ETs)	Total (N = 254)
Men	6 (13.6%)	18 (40.9%)	20 (45.5%)	44
Women	50 (23.8%)	108 (51.4%)	52 (24.8%)	210

## Data Availability

The data presented in this study are available on request from the corresponding author.
